# Nudix Effectors: A Common Weapon in the Arsenal of Plant Pathogens

**DOI:** 10.1371/journal.ppat.1005704

**Published:** 2016-08-11

**Authors:** Suomeng Dong, Yuanchao Wang

**Affiliations:** 1 College of Plant Protection, Nanjing Agricultural University, Nanjing, China; 2 The Key Laboratory of Integrated Management of Crop Diseases and Pests, Ministry of Education, Nanjing, China; The Sainsbury Laboratory, UNITED KINGDOM

## Overview

Plant pathogens secrete a variety of unique and highly specialized effectors to manipulate host immunity. Only a small proportion of the conserved effector genes are present in the genomes of pathogens across kingdoms, providing us with good opportunities to study plant immunity and disease mechanisms. Nucleoside diphosphate-linked moiety X (Nudix) effectors, a group of secreted proteins containing the Nudix hydrolase domain, have been identified in a broad range of pathogens including bacteria, oomycetes, and fungi. Nudix effectors have been validated as pathogenesis players in a few host–pathogen systems, indicating that an important virulence strategy might be shared by distinct plant pathogens. Although progress has been made, many questions remain yet unsolved. Here, we summarize our current knowledge of Nudix effectors. We will also provide thoughts and discussions for the next phase of research.

## What Are Nudix Proteins and Nudix Effectors?

The nucleoside diphosphate-linked moiety X (Nudix) hydrolases constitute a superfamily of proteins that are ubiquitous in both prokaryotic and eukaryotic organisms [1]. The superfamily members are normally characterized by the presence of a conserved Nudix box (GX5EX7REUXEEXGU, U is usually Ile, Leu, or Val, X is any residue), which catalyzes degradation of nucleoside diphosphate-X (NDP-X) to nucleoside monophosphate (NMP) and phosphate-X (P-X) [1]. Studies in model organisms showed that Nudix proteins perform a variety of functions to sense and modulate levels of their substrates like nucleotide sugars, deoxyribonucleoside triphosphate (dNTPs), and capped mRNAs to maintain proper cellular processes and physiological homeostasis [2,3]. An appealing observation to plant pathologists is that Nudix proteins act as regulators in plant immunity. *Arabidopsis* AtNUDT7, a well-studied Nudix protein, was initially identified as a pathogen-responsive gene [4,5]. *Atnudt7* knockout mutants showed constitutive expression of defense-related genes and accumulation of higher levels of salicylic acid, resulting in enhanced basal defense against bacteria pathogen *Pseudomonas syringae* and oomycete pathogen *Hyaloperonospora parasitica* [4]. Investigations demonstrated that AtNUDT7 negatively regulates EDS1 signaling pathway, one of the key components in plant immunity [6]. Interestingly, AtNUDX8, another Nudix protein, was a positive regulator of plant defense. Knockout mutants displayed repressed salicylic acid signaling and significantly enhanced susceptibility to pathogens [7]. Therefore, plant Nudix proteins appear as important players in the plant–pathogen battlefield.

Effector proteins, secreted by plant pathogens to manipulate host defense systems, are good probes for dissecting plant immunity. A special group of Nudix proteins characterized by a secretory signal peptide preceding the Nudix domain, designated as Nudix effectors, has been identified in a variety of plant pathogens. The first nudix effector was Hpx26, a novel type III secretory system (T3SS) effector that was screened by using a transposon-based system in phytobacteria *Ralstonia solanacearum* [8]. The first eukaryotic Nudix effector, PsAvr3b, was identified as an avirulence effector in a soybean root rot oomycete pathogen through a map-based cloning approach [9]. Thus, different approaches in distinct plant–pathogen systems have independently identified Nudix effectors.

## Does a Broad Range of Pathogens Recruit Nudix Effectors?

**Fig 1:**
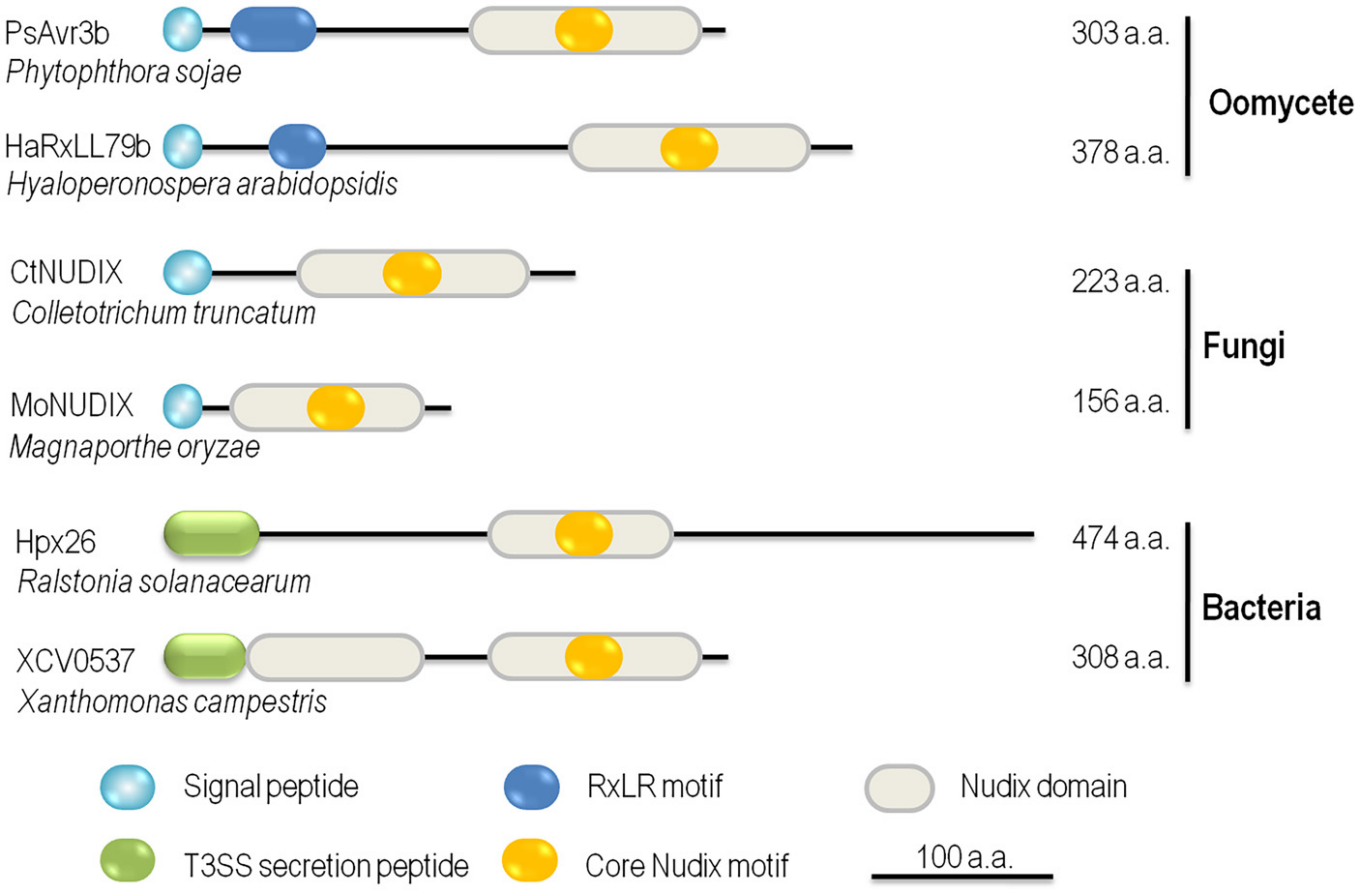
Nudix effectors from pathogens across the kingdoms. Six reported Nudix effectors from pathogenic oomycetes, fungi, and bacteria are presented. PsAvr3b is an effector from soybean *Phytophthora* root rot pathogen isolate P6497 [9]. HaRxLL79b is an *in planta*-induced RXLR-like effector identified in *Arabidopsis* downy mildew *H*. *parasitica* isolate Waco9 [19]. Nudix effectors CtNUDIX and MoNUDIX are derived from two hemibiotrophic fungi: a broad host range *Colletotrichum* pathogen and the rice blast causal agent *Magnaporthe oryzae*, respectively [15]. Both Hox26 and XCV0537 are type III secretory effectors from vascular wilt bacteria and pepper or tomato leaf spot bacteria [8,11]. The effectors are drawn to scale, with the scale bar provided at the bottom of the figure.

Nudix effectors have been reported in plant pathogenic oomycetes, fungi, and bacteria so far, suggesting that this category of effectors might be important virulence components in the "toolbox" of plant pathogens. We illustrate a schematic view of six representative Nudix effectors varied in structure and size (Fig 1). The expression of Hpx26 effector from *R*. *solanacearum* is orchestrated with many other T3SS effectors and the secretion of Hpx26 was verified [8,10]. In *Xanthomonas campestris* pv. *vesicatoria*, T3SS Nudix effector XCV0537 was identified [11]. Our BLAST search revealed that the Hpx26 and XCV0537 homologs are widely presented in different *Ralstonia* and *Xanthomonas* species. HopAG1, a T3SS effector with putative Nudix domain in *Pseudomonas syringae* pv. *syringae* isolate B728a, was previously reported [12], whereas HopAG1 orthologs were pseudogene in many *P*. *syringae* isolates, including DC3000 [13,14]. Moreover, a Nudix effector, namely CtNUDIX, was experimentally validated in fungal *Colletotrichum truncatum* [15]. Genome sequencing of *Colletotrichum* pathogens resulted in the identification of one putative Nudix effector from the *C*. *graminicola* genome, three from *C*. *higginsianum*, two from *C*. *orbiculare*, and two from *C*. *fructicola*. The genome of *Magnaporthe oryzae*, the rice blast fungus, also revealed two duplicate copies of putative Nudix effectors [15,16]. However, BLAST searches did not yield CtNUDIX homologous proteins in the genomes of selected phytopathogenic fungi, including *Puccinia graminis*, *Ustilago maydis*, *Sclerotinia sclerotiorum*, and *Verticillium dahliae* [15]. In oomycetes, PsAvr3b employs a conserved Arg-X-Leu-Arg (RXLR) host-targeting motif, which is responsible for effector entry into host cells [17,18]. Furthermore, both *Phytophthora ramorum* and *Phytophthora capsici* genomes encode three putative RXLR-type Nudix effectors. In the Irish potato famine pathogen *Phytophthora infestans*, however, Nudix effector gene seems to have undergone gene expansion—a total of five RXLR-type and two non-RXLR-type Nudix effectors have been identified [9,15]. Two RXLR-like Nudix effectors, HaRxLL79b/c, were highly induced during infection of *H*. *parasitica* [19]. However, we did not harvest Nudix effectors in the necrotrophic oomycete *Pythium ultimum* and the fish pathogen *Saprolegnia parasitica*.

## What Role Does the Nudix Effector Play during Host–Pathogen Interactions?

Although a broad range of plant pathogens employs Nudix effectors, the biological roles of these Nudix effectors remain poorly understood. Independent studies reported that T3SS Nudix effectors from *X*. *campestris* and *R*. *solanacearum* are induced during infection of host plants like many other T3SS effectors [8,11]; however, the biological functions of bacterial Nudix effectors have not been reported.

**Fig 2:**
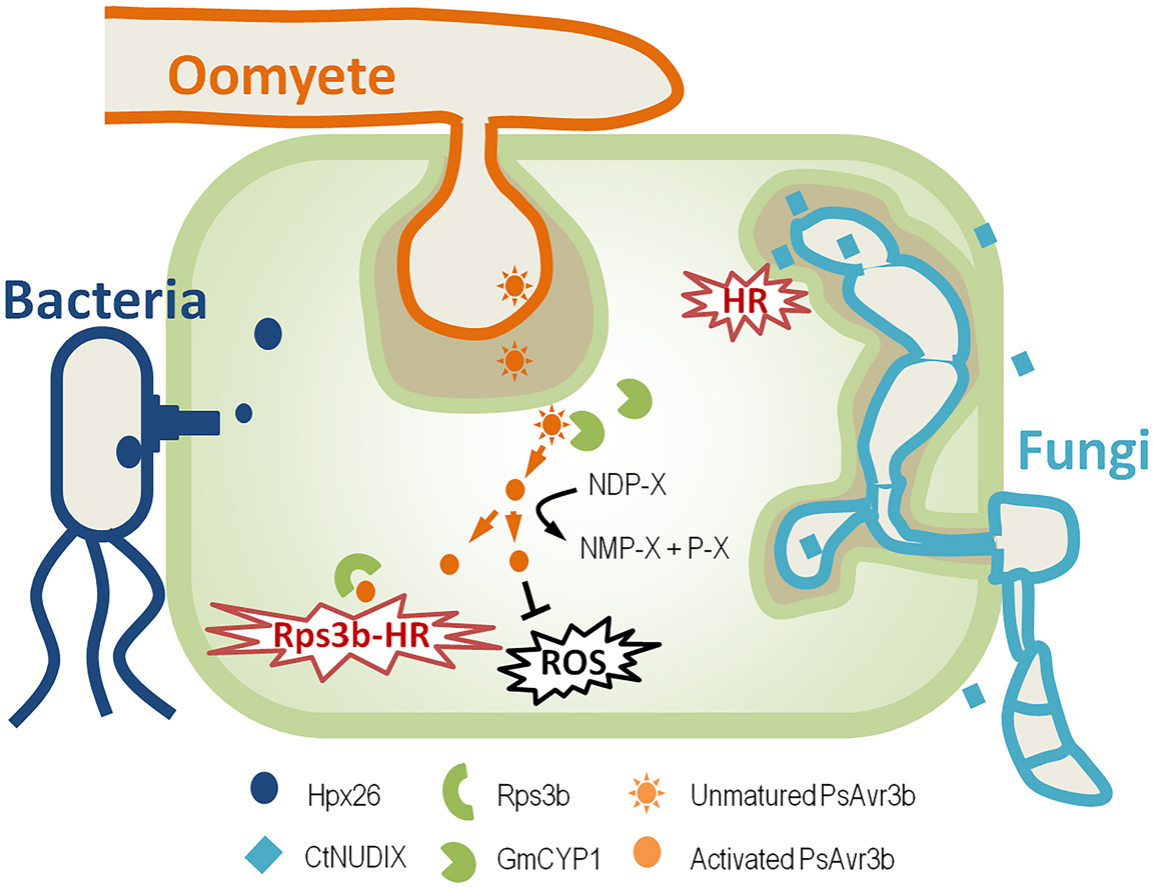
Schematic representation of the mode of action of Nudix effectors from distinct plant pathogens. Like many other bacteria effectors, Hpx26 from *R*. *solanacearum* is induced during infection and is secreted in a T3SS-dependent manner [8,10]. Fungal Nudix effector CtNUDIX from *C*. *truncatum* functions in plant apoplast or on plant plasma membrane. It elicited hypersensitive response (HR)-like cell death and is exclusively expressed during the late biotrophic stage [15]. *P*. *sojae* RXLR-type Nudix effector PsAvr3b is expressed at early infection stage and is delivered into host cell, where it recruits plant cyclophilin GmCYP1 to maturate itself. Activated PsAvr3b is able to block host ROS, and it also triggers soybean Rps3c-mediated HR [9,23].

The most well-studied Nudix effector in oomycete pathogens is PsAvr3b from *Phytophthora sojae* (Fig 2). Ectopic expression of PsAvr3b in tobacco increased plant susceptibility to *Phytophthora*, with significantly reduced accumulation of reactive oxygen species (ROS) around invasion sites, indicating a virulence function of PsAvr3b [9]. Furthermore, biochemical assays demonstrated that PsAvr3b is an ADP-ribose/NADH pyrophosphorylase. Abolishing the enzymatic activity significantly impaired PsAvr3b virulence, but not avirulence activity. This is consistent with the natural variations data that both PsAvr3b avirulent and virulent proteins exhibit Nudix activity. These data indicate that although PsAvr3b Nudix hydrolase activity is important for virulence, but it is not required for the recognition by host immune receptor Rps3b [9]. Both PsAvr3b and plant susceptibility regulator AtNUDT7 prefer similar substrates in vitro; we therefore assumed that PsAvr3b mimics plant negative immune regulator to impair host immunity.

In contrast, the Nudix effector CtNUDIX from *C*. *truncatum* functions differently (Fig 2). CtNUDIX elicited hypersensitive response (HR)-like cell death in tobacco leaves and is exclusively expressed during the late biotrophic stage [15]. CtNUDIX, lacking a signal peptide or a Nudix motif, failed to induce cell death in tobacco; meanwhile, it localized on cell plasma membrane. Therefore, CtNUDIX might primarily hydrolyze extracellular targets or plasma membrane components [15]. Overexpression of CtNUDIX effector in *C*. *truncatum* and *M*. *oryzae* elicits HR-like responses in the infection site, resulting in incompatibility in the host plant. Thus, it is speculated that CtNUDIX switch the fungal lifestyle from biotrophy to necrotrophy. However, the substrates and virulence mechanisms of CtNUDIX have yet to be determined.

## Do Nudix Effectors Require Host Factors to Function?

In humans and yeast, many Nudix proteins like Dcp2 are associated with protein complexes to perform functions [20–22]. Recently, it has been reported that the Nudix effector PsAvr3b recruits a host prolyl-peptidyl isomerase (PPIase) cyclophilin protein to modify itself properly for full function [23]. It is shown that PsAvr3b interacts directly with soybean cyclophilin GmCYP1 in vivo and in vitro, and GmCYP1 in turn activates the hydrolase activity of PsAvr3b in a PPIase activity-dependent manner, possibly by isomerizing PsAvr3b into a proper conformation. The substitution of PsAvr3b putative Glycine-Proline (GP) motif, which is a known binding site in cyclophilin substrates, impaired the interaction of PsAvr3b with GmCYP1. Consequently, this mutant can no longer be activated by GmCYP1 and is unable to promote *Phytophthora* infection or trigger Rps3b-mediated soybean defense response [23]. In summary, this report demonstrated that the cyclophilin protein activates the enzymatic activity of PsAvr3b as a "helper" in host cells to promote PsAvr3b function. Whether other Nudix effectors require additional host factors for function is of interest for further investigation.

## What Is the Future Direction of Nudix Effector Research?

The most striking question that remains unclear is the biological functions of Nudix effectors. Beside conserved Nudix domain, the structures and sequences vary greatly among Nudix effectors from different kingdoms, indicating biological functions of these effectors might be different. Although in vitro preferred substrates of PsAvr3b were determined, the natural substrates and the in vivo Nudix enzymatic functions for Nudix effectors as well as their relationship with plant immunity remain poorly understood. Secondly, the protein localization pattern usually provides good information for effector activity. Nudix proteins localize in distinct subcellular components including P-body, peroxisome, and cytosol, which are important to their functions [22,24]; however, little is known for Nudix effectors. Scanning Nudix effectors did not reveal any robust subcellular localization signature; cell biology approaches will be required to investigate Nudix effector subcellular localization. To investigate Nudix effector functions further, a third alternative is to identify binding proteins. Whether these effectors recruit host helper protein to function properly, or what host immune proteins do effectors target to, deserves further exploration.

Interestingly, Nudix proteins play important roles in animal and human pathogens. A Nudix hydrolase gene *YSA1* in the human pathogen *Cryptococcus neoformans* was found to modulate the oxidative stress response and susceptibility to chemical drugs [25]. In the zoonotic pathogen *Leptospira*, nudix protein InvA is required for pathogen virulence [26]. Interestingly, we identified putative secreted Nudix proteins in the *C*. *neoformans* and *Cryptococcus gattii* genomes through BLAST searches (S1 Text); the presence of expressed sequence tag (EST) sequence was also detected in National Center for Biotechnology Information (NCBI) database. However, studies on these genes have not been reported to the best of our knowledge. Nevertheless, the biological functions of these effectors in manipulating animal and human immunity are interesting questions for future examination.

In summary, emerging reports that Nudix proteins play important roles during infection, as well as the presence of Nudix effector genes in pathogens across kingdoms, shed light on an important virulence mechanism shared by plant pathogens and potentially by animal pathogens as well. Why do distinct pathogens independently evolve Nudix effectors? From where do these Nudix effectors evolve? How do pathogens utilize this group of effectors? Further studies should combine biochemical analyses with genetics and biophysical studies to address these questions to gain a thorough understanding of the biology of Nudix effectors.

## Supporting Information

S1 Text Putative secreted Nudix proteins in *Cryptococcus* pathogens. XP_012052811.1 and KIR63716.1 are National Center for Biotechnology Information (NCBI) accession numbers. The bold sequences highlighted in yellow represent signal peptides (predicted by both SignalP 2.0 and SignalP 3.0). The bold italicized sequences highlighted in red are predicted Nudix motifs (based on a conserved domain search).
(DOCX)
