# Correction: Nudix Effectors: A Common Weapon in the Arsenal of Plant Pathogens

**DOI:** 10.1371/journal.ppat.1005970

**Published:** 2016-10-13

**Authors:** Suomeng Dong, Yuanchao Wang

There is an error in how protein XCV0537 is referred to in the fourth sentence of the "Does a Broad Range of Pathogens Recruit Nudix Effectors?" section and in the penultimate sentence of the Figure 1 legend.

In the fourth sentence of the "Does a Broad Range of Pathogens Recruit Nudix Effectors?" section, the word "putative" should be added so that the sentence instead reads:

"In *Xanthomonas campestris* pv. *vesicatoria*, the putative T3SS Nudix effector XCV0537 was identified [11]."

In the Figure 1 legend, the penultimate sentence should instead read:

"Hpx26 is a type III secretory effector from vascular wilt bacteria [8] and XCV0537 is a putative type III secretory effector from pepper or tomato leaf spot bacteria [11]."
