# Supplementary material for: Nudix Effectors: A Common Weapon in the Arsenal of Plant Pathogens
Source: PLoS Pathog. 2016 Aug 11;12(8):e1005704. doi: 10.1371/journal.ppat.1005704 (PMC4981494; doi:10.1371/journal.ppat.1005704)
Supplement: S1 Text — XP_012052811.1 and KIR63716.1 are National Center for Biotechnology Information (NCBI) accession numbers. The bold sequences highlighted in yellow represent signal peptides (predicted by both SignalP 2.0 and SignalP 3.0). The bold italicized sequences highlighted in red are predicted Nudix motifs (based on a conserved domain search). (DOCX) [file ppat.1005704.s001.docx]

**S1 Text. Putative secreted Nudix proteins in *Cryptococcus* pathogens.**

> XP_012052811.1 (*Cryptococcus neoformans* var*. grubii* H99)

**MVASTPTETPSTALLSLIHSLRA**LPTRLIQSPPTQPRRASVAIIIRLRPAEDLVFEGHEPEGWTGNVVSREDWGEGLELEDFMKLSWVNHPNTVPEILFIRRASPSSLPPPGAHHRWASHIAFP***GGRQEPDDQSAYYTALRETWEEIGI***DLAEKEFLNVGRLDEREVTTSLGKRLLMILSPFVFIQTTPISPAPELQATEISSVHWVPLSLLTPPFSPSRWSHVEIDVSTRLSPRNKFVRWCLRNLIGKMKFGCLLLPDEPTVTAENFDPLDFDETIEGSGSWTDAANGSRFLRLWGLTLGMTLDLISHHPSAPSKLFAEGQNLTTSQPSTPVMDYNPQLAPRTPVTTHSTFEDQWEAARKVLAEEEKNRARANEKAAQGRRRRGVGPYMTAVFPRFTYPDVNFWIWVFSRRYRQVLKSWELSAIGPSRAADRRINWSGQALATFYTAVRQALVVTLIIRALGLGVGLAGVGYLVFKAMGGGEL*

>KIR63716.1 (*Cryptococcus gattii* CA1873)

**MVASTPTETPSTALLSLIHSLRA**LPTRLIQSPPTQPRRASVAIIIRLRPAEELVFEGHEPEGWTGDVISREDWGEGLELEDFMKLSWVNHPNTVPEILFIRRASPASSPPPGAHHRWASHIAFP***GGRQESDDQSAYYTALRETWEEIGI***DLAEKEFLNVGRLDEREVTTSLGKRLLMILSPFVFIQTTPISPTPELQAAEISSVHWVPLSLLTPPFSPSRWSHVEIDVSTRLSPRNKFVRWCLRNLIGKMKFGCLLLPDEPAVTAEDFDPFGFDETVEGSGSWTNAADGSRFLRLWGLTLGMTLDLISHHPSAPSKLLAEGQNLITSQPSTPVMDYKPQLAPRTPVTTHSTFEDQWEAARKALVEEEKNRAKASEKATQSRRRRGNSSAVFPRFTYPDVNFWIWIFSRRYRQVLKSWELSAIGPSRAADRRINWSGQALATFYTAVRQALVVTLIIRALGVGVGLAGVGYLVFKAMGGGEL*

XP_012052811.1 and KIR63716.1 are National Center for Biotechnology Information (NCBI) accession numbers. The bold sequences highlighted in yellow represent signal peptides (predicted by both SignalP 2.0 and SignalP 3.0). The bold italicized sequences highlighted in red are predicted Nudix motifs (based on a conserved domain search).
